# When growth models are not universal: evidence from marine invertebrates

**DOI:** 10.1098/rspb.2013.1546

**Published:** 2013-10-07

**Authors:** Andrew G. Hirst, Jack Forster

**Affiliations:** School of Biological and Chemical Sciences, Queen Mary University of London, Mile End Road, London E1 4NS, UK

**Keywords:** growth, invertebrates, marine, metabolic theory of ecology, West Brown Enquist

## Abstract

The accumulation of body mass, as growth, is fundamental to all organisms. Being able to understand which model(s) best describe this growth trajectory, both empirically and ultimately mechanistically, is an important challenge. A variety of equations have been proposed to describe growth during ontogeny. Recently, the West Brown Enquist (WBE) equation, formulated as part of the metabolic theory of ecology, has been proposed as a universal model of growth. This equation has the advantage of having a biological basis, but its ability to describe invertebrate growth patterns has not been well tested against other, more simple models. In this study, we collected data for 58 species of marine invertebrate from 15 different taxa. The data were fitted to three growth models (power, exponential and WBE), and their abilities were examined using an information theoretic approach. Using Akaike information criteria, we found changes in mass through time to fit an exponential equation form best (in approx. 73% of cases). The WBE model predominantly overestimates body size in early ontogeny and underestimates it in later ontogeny; it was the best fit in approximately 14% of cases. The exponential model described growth well in nine taxa, whereas the WBE described growth well in one of the 15 taxa, the Amphipoda. Although the WBE has the advantage of being developed with an underlying proximate mechanism, it provides a poor fit to the majority of marine invertebrates examined here, including species with determinate and indeterminate growth types. In the original formulation of the WBE model, it was tested almost exclusively against vertebrates, to which it fitted well; the model does not however appear to be universal given its poor ability to describe growth in benthic or pelagic marine invertebrates.

## 1. Introduction

The increase in an individual's body mass through time can be described through a growth function. The trajectory of such mass change is fundamental, as body size determines many life-history traits, such as rates of fecundity [1,2], mortality [3] and population increase [4]. However, there is still much debate as to the best way of modelling growth through ontogeny [5–8]. Clearly, being able to quantitatively describe such trajectories through the ontogeny of animals, and to define whether taxa share a single universal response or indeed differ in this fundamental respect, are critical to our understanding and ability to formulate mechanistic approaches to their metabolism and life history. Such models also add to our ability to make predictions of rates and their pattern across nature. Historically, different growth models have been used for different taxa. Sigmoidal functions, such as the von Bertalanffy growth equation, have been commonly applied to many vertebrates [9–11]. Invertebrate growth, on the other hand, has often been modelled using exponential [12–14] or power (allometric) functions [6]. However, von Bertalanffy-type growth equations have been applied to a number of invertebrate groups recently, including various gelatinous taxa [15], mussels [16] and benthic invertebrates [17]. The range of models applied to invertebrates, and the apparent inconsistency in their choice, highlights a lack of understanding of which models are most suitable and when they should be applied. A critical appraisal of growth functions is required in order to determine whether any single model is universal, and indeed which models are the most appropriate.

Developing unifying approaches to explain the life history and physiology of organisms, including their growth, can aid in understanding the underlying drivers and mechanisms. In recent years, this has included application of the metabolic theory of ecology (MTE) to a wide range of physiological and population processes [5,18,19], while diverse aquatic environments can provide important test cases [20,21]. The MTE has also been applied to the allocation of metabolic energy to determine the increase in mass in individuals [5]. This has the same mathematical form as the von Bertalanffy [22] growth equation:1.1
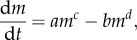
where *a* and *b* are determined from cellular properties, *c* is assumed to be 3/4, *d* assumed to be 1, *m* is mass and *t* is time [5,23]. In the original formulation of the model by von Bertalanffy [22], the term *am^c^* represents anabolic processes (the building up of material), whereas *bm^d^* represents catabolic processes (the breaking down of material). The West Brown Enquist (WBE) model differs from the von Bertalanffy formulation in that *c* and *d* have defined set values, while *a* and *b* are directly calculated from fundamental cellular properties. Equation (1.1) forms the basis of the WBE model, which has been purported to describe the growth of many diverse species and is given as1.2
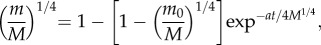
where *M* = asymptotic mass, *m*_0_ = initial egg or progeny mass and *t* = time.

**Figure 1.**
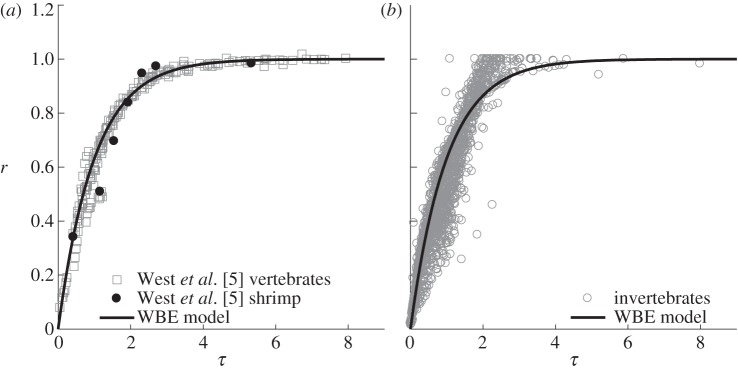
Plots of dimensionless mass (*r*) against dimensionless time (*τ*). (*a*) Data from the original West *et al.* [5] paper, containing mostly vertebrate examples (12 of 13 species). The single invertebrate species included in their study is highlighted in black. (*b*) Data from this study for marine invertebrates. In both plots, a comparison with the WBE model, equivalent to the universal growth curve 1−exp^−*τ*^ [5], is given. *r* is the dimensionless mass variable (*m*/*M*)^1/4^; *τ* is the dimensionless time variable (*at*/4*M*^1/4^) − ln(1−(*m*_0_/*M*)^1/4^). Parameters are defined in equation (1.2).

The WBE model is based on fundamental biological processes, and hence if, when tested, it proves to be widely applicable, this may provide important insights into growth based on basic cellular properties. As West *et al.* [5, p. 628] state: ‘Several equations have been proposed to describe ontogenetic growth trajectories for organisms justified primarily on the goodness of fit rather than on any biological mechanism’. However, it is also important that models should fit empirical data well, and preferably better than less complex equation forms. In their original growth paper, West *et al.* [5] fitted empirical data to their model for a range of species and compared them (using dimensionless time and mass axes). Their analysis, however, mostly contained data for vertebrate species (12 of the 13 species tested were vertebrates). The single species of invertebrate, the marine shrimp *Mysis mixta*, appears on visual inspection to have a poorer fit to the WBE model (figure 1). Given this, and that growth in many marine invertebrates has commonly been modelled using an exponential function [24,25], a systematic test to establish which models are best is needed. Marine invertebrates are especially useful because they represent a wide diversity of phyla and life-history types within an important ecosystem, and present a good test for any general models.

We address the following questions in this study. (i) How well does the WBE model describe the ontogentic growth trajectories of marine invertebrates? (ii) Which model (power, exponential or WBE) has the greatest ability to describe growth? (iii) Does the best model vary between taxa, or does a single model best describe ontogenetic growth in marine invertebrates?

## 2. Material and methods

We searched for publications that included laboratory measurements of the increase in mass of an organism as a function of time (i.e. through ontogeny). Data were identified using the ISI Web of Knowledge. Search terms used were ‘marine AND growth AND (development OR larva* OR ontogen* OR “life history”)’. Individual journals were also searched separately (e.g. *Aquaculture*, *Journal of Crustacean Biology*, *Journal of Plankton Research*, etc.). Data for individual body mass versus time were collected, with time set as zero at the initial life stage: data were included only where time was measured from either the egg at the point of hatching or progeny at the point of birth. Mass units were included as wet, dry, carbon or ash-free dry weights, or alternatively as volume. In all cases, we converted these values to dry weight (see the electronic supplementary material, appendix S1). We also collected data for *m*_0_: the mass of the initial egg (or first larval) stage and asymptotic mass *M*. Wherever these data were not available in the original study, they were collected from alternative literature sources (see electronic supplementary material, appendix S1). Data were divided into 15 separate taxa: Amphipoda, Anostraca, Anthozoa, Appendicularia, Cephalopoda, Chaetognatha, Copepoda, Ctenophora, Decapoda, Doliolida, Echinodermata, Euphausiacea, Gastropoda, Nematoda and Polychaeta. The experimental temperature and the sex were also noted for each species. Data were accepted only where food was provided ad libitum, and the feeding regime was recorded. We limited our data inclusion to laboratory situations where conditions were controlled and maintained near constant; this allowed potential effects of variable food quality/quantity and temperature on ontogenetic growth rates to be excluded.

Having collected data for change in mass against time, *m*_0_ and *M*, we compared the marine invertebrate data against the WBE model [5]. Data were divided into sets; each dataset was represented by a single species of a single sex at a single temperature in a single study, hence some studies gave more than one dataset. The WBE model was iteratively fitted to each set using SigmaPlot v. 10.0 and values for parameter *a* were determined by applying a rearrangement of equation (1.2):2.1
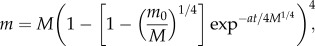
where *m* = dry mass (mg) at time *t*, *m*_0_ = progeny dry mass (mg) and *M* = asymptotic maximum dry mass (mg). Using these data, we determined values for dimensionless mass ratio *r* as (*m*/*M*)^1/4^ and dimensionless time variable *τ* as (*at*/4*M*^1/4^)−ln(1−(*m*_0_/*M*)^1/4^) [5]. Data that did not cover a significant portion of the ontogenetic mass increase, defined as *r* < 0.1, were removed from any further analysis. Those datasets that met this criterion were plotted against the WBE model, represented by the universal growth curve 1 − exp^−*τ*^, in order to determine how well this curve modelled marine invertebrate growth. We calculated residuals for data versus the WBE model and plotted these against dimensionless time *τ*.

The power, exponential and WBE equation forms were compared with each dataset (a single species of a single sex at a single temperature in a single study) to determine which of these best described growth. We calculated the residual sums of squares for each equation form by iteratively fitting each in turn using SigmaPlot v. 10.0. Which model fitted each dataset best was determined using an information theoretic approach [26,27]; in all cases we restricted this test to datasets where the number of data points for mass versus time was greater than 5. Akaike weights (*ω*_*i*_) were calculated for each model within each dataset (see the electronic supplementary material, appendix S2), and mean Akaike weights were subsequently calculated for each taxon. Further, the best model was selected for individual datasets where there was sufficient strength of evidence to reject the other two models. This was defined as the best model having an Akaike weight greater than 10 times that of each of the other two models, following the methods of Royall [28]. The percentage frequency of best models (i.e. 100 × number of times equation form was best model/total number of datasets with a single best-fit model) were compared for each taxon. We tested for any effects of pelagic versus benthic lifestyles by comparing best-fit models for each of these two groupings separately.

## 3. Results

**Figure 2.**
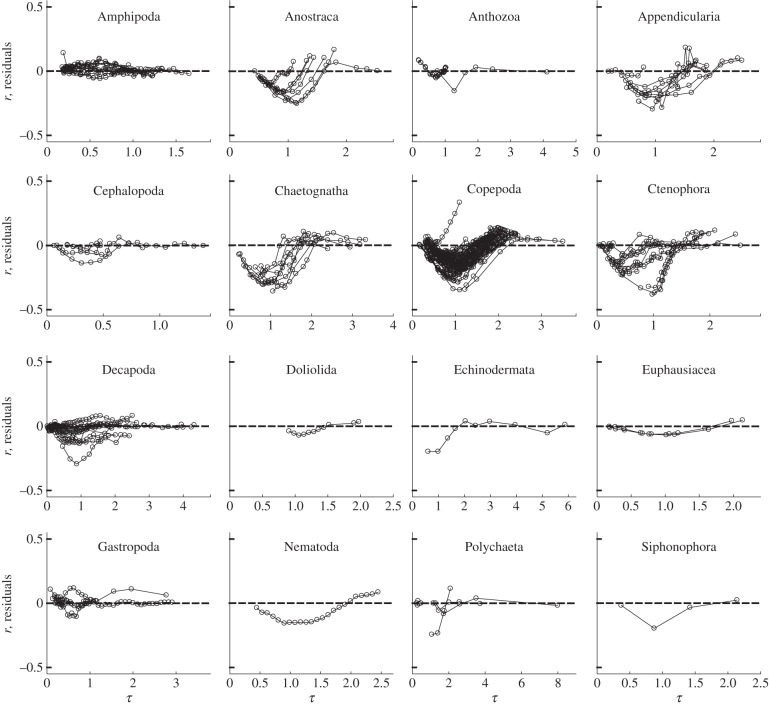
Residuals of the dimensionless mass variable *r* for marine invertebrate taxa when compared against that predicted by the WBE model (figure 1). Residuals for each point are plotted against the dimensionless time variable *τ*. Connected points represent a single dataset defined as species data separated by study, temperature and sex. See figure 1 for definitions.

We collected 139 datasets for 58 different marine invertebrate species. The largest number of datasets were for Copepoda (52 datasets) and Decapoda (20 datasets), with all other taxa containing fewer than 15 datasets. Initial comparisons of the growth of marine invertebrates against the WBE model showed that the data were not modelled well by this curve (figure 1). When *τ* in the WBE model was less than 2 (i.e. where approx. 85% of maximum mass is reached; figure 1*b*), over two-thirds of the data (1014 of 1428 data points) fell below the WBE model prediction. When *τ* was greater than 2, the majority of data (113 of 144 data points) fell above the WBE model prediction. This systematic divergence of the empirical data from the model prediction suggests a problem. Although in the original paper of West *et al.* [5] the growth data used to examine the WBE model fitted much better (figure 1*a*), in their study, 12 of 13 species included were vertebrates. From our taxon-specific residual analysis for marine invertebrates (figure 2), it can be seen that the divergence from the WBE model (figure 1) is driven by the poor fit in a number of taxa, specifically Anostraca, Appendicularia, Chaetognatha, Copepoda and Ctenophora. The Amphipoda, on the other hand, fitted the WBE model well (figure 2).

**Figure 3.**
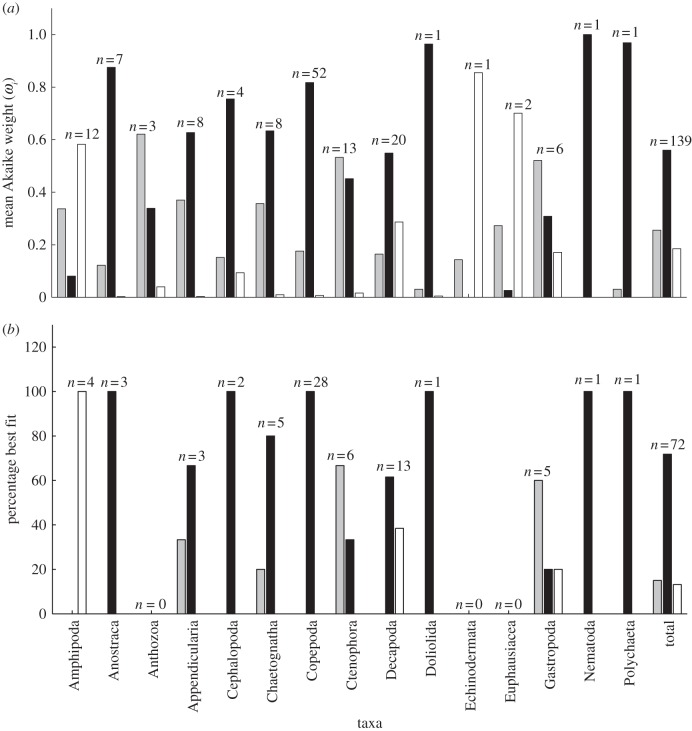
(*a*) Comparison of the best-fit models (power, exponential or WBE), calculated using Akaike weights (see electronic supplementary material, appendix S2). Mean Akaike weights are determined by the sum of *ω*_*i*_ for each model for each taxon divided by the number of datasets for each taxon. (*b*) Frequency of best fit for each model type (%) for those datasets where one of the three models was deemed better than the other two. WBE, West Brown and Enquist model [5]; *n*, the number of separate growth curves for each taxon. Grey bars represent power model, black bars represent exponential model and white bars represent WBE.

Using an information-theoretic approach, we found that an exponential model was most often the best fit to the marine invertebrate data (figure 3). Across all 139 datasets, the mean Akaike weight for the exponential model was 0.56, versus 0.25 for the power model and 0.18 for the WBE model (figure 3*a*). Further, when we examined only those datasets where one model was deemed better than the other two (see Material and methods), we found the exponential form to be best in 53 of the 72 datasets; this compares with 9 for the power model and 10 for the WBE model (figure 3*b*). Of all the taxa, the WBE model performed well in the Amphipoda (figure 3*b*), supporting the findings presented for the WBE model residuals (figure 2). The power model performed well in Ctenophora and Gastropoda. The exponential model, on the other hand, performed well in nine of 15 taxa. These results are not simply driven by an exponential model being a better fit when growth data were available through only a limited part of ontogeny. In the Copepoda, mass versus time data were comprehensive, and distributed from egg to adult stages, yet the exponential model once again provided the best fit. This was also the case for the Appendicularia and Cephalopoda data, where the exponential model gave a better fit than the other models (figure 3; see the electronic supplementary material, appendices S1 and S2).

We tested for any effects of pelagic versus benthic lifestyles by comparing best-fit models and found no significant difference between the relative frequency of the best fit of these models: in both benthic and pelagic organisms, the exponential model fitted best, while power and WBE models were best fit in a minority of cases.

**Figure 4.**
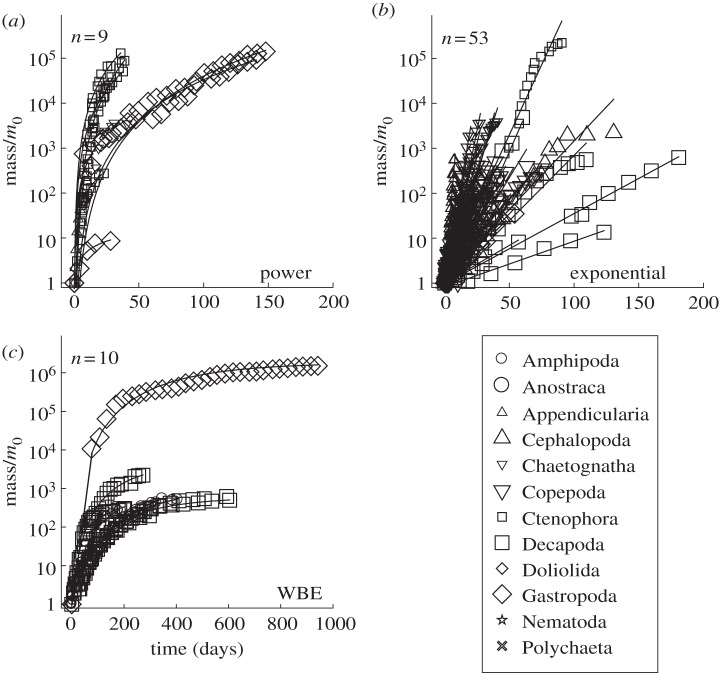
Normalized mass (mass divided by progeny mass, *m*_0_) on a log scale as a function of time (days) for marine invertebrate taxa. Growth trajectories are shown by the model ((*a*) power, (*b*) exponential and (*c*) WBE) that is the best fit, for datasets where one model was deemed better than the other two, as determined from Akaike weights. *n* is the number of cases in which each particular model was best.

We examined mass–time data in detail for those datasets in which a single model was defined as the best fit (figure 4). The majority of these were best described by an exponential model, giving a linear response on a log mass versus time plot. Most marine invertebrate species do not show the marked curvature in their mass versus time trajectory, forming a plateau, as the WBE suggests.

## 4. Discussion

Within this study, which includes a wide range of pelagic and benthic marine invertebrates, we found that when a single growth model could be classified as being best (when its Akaike weight was greater than 10 times that of each of the two other models), it was most often the exponential form (approx. 73% of cases) that met this criterion. By contrast, the power model was the best fit in just approximately 13% of cases and the WBE model in approximately 14% of cases. The WBE model tended to overestimate mass during the early stages of ontogeny but underestimate mass in late ontogeny (figures 1 and 2). In the original model formulation, West *et al*. [5] (figure 1) compared the model with empirical growth data from mostly vertebrates, and it was found to fit well. Data for marine invertebrates, however, appear to commonly follow a very different growth trajectory (figure 1), suggesting generalization of their model to these taxa to be inappropriate. Support for the WBE model was, in part, based upon it containing a proximate mechanism. Furthermore, it was suggested that, because many different growth models provide good fits to mass versus time data, there is little basis for choosing among these models on statistical grounds [29]. This second point does not agree with the findings here: we suggest that the use of an information-theoretic approach does distinguish between these models on statistical grounds, and in the majority of cases for marine invertebrates does not support the application of the WBE model.

Individual growth is a highly plastic trait, varying with temperature [30,31], food abundance [32] and predation [33]. A model to fit change in mass with time needs to be able to account for such differences or be simple enough to incorporate further parameters to explain such factors. We suggest the application of the WBE growth equation to invertebrate taxa should be conducted with caution, given the highly variable nature of growth and the poor fit it provided for marine invertebrate data. Using a simpler model, for example an exponential, may often be more appropriate, and does allow the incorporation of factors such as temperature, food and predation more easily into any attempt to model growth rates in the natural environment.

Why does the WBE model perform poorly with respect to the majority of marine invertebrate growth data? Marine invertebrate taxa have very different life-history strategies than vertebrates. Similar to vertebrate species, mortality rates in marine invertebrates are high in early ontogeny [34]. However, in taxa such as Anostraca [35], Appendicularia [36] and Copepoda [37], which clearly exhibit exponential growth, mortality rates remain high throughout the life cycle, owing to environmental or predation pressures. This is not the case in many vertebrate species, where mortality rates tend to decrease significantly through ontogeny [38,39]. This puts a premium on rapid growth in marine invertebrates, which may explain their exponential form: species maximize their fitness by growing to maturity as rapidly as possible.

Another potential factor to explain the poor fit of the WBE may be that of determinate versus indeterminate growth [8,40]. Determinate growers reach a fixed adult size, devoting any surplus energy into reproduction beyond the point of maturity. Indeterminate growers show a gradual decline in energy converted to somatic growth and an increase in that devoted to reproduction. These two strategies produce different shape to body size change through ontogeny. In determinate growth, body size should reach a mature level, at which point the increase in size stops. Indeterminate growers, on the other hand, should show a gradual decrease in trajectory post-maturity [8]. Given that the von Bertalanffy equation should model lifetime body size [8], the WBE derivation of the equation may not model growth of marine invertebrates well because the datasets do not all contain body size data throughout the entire lifetime. However, we may expect any taxa that display determinate growth to be well described by the WBE model, as asymptotic body size is equivalent to body size at maturity. We do not find this to be the case; of the 15 taxa included, three show determinate growth—Appendicularia, Copepoda and Nematoda. Yet all three of these were better modelled by an exponential model (figure 3). This is the case despite the majority of appendicularian data and all the copepod data including mass data to maturity. Further, in some indeterminate growers where data were available up to and beyond maturity (e.g. the cephalopod *Euprymna scolopes* and the ctenophore *Pleurobrachia pileus*; electronic supplementary material, appendices S1 and S2), the WBE model again performed poorly.

Given that the exponential equation was best at modelling changes in mass with time, this suggests that growth in many marine invertebrate taxa is best modelled using an equation of the form4.1
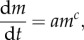
where *c* = 1. Hence, within the species and taxa we test, the exponent *c* is more commonly found to fit a value of 1 than it is 0.75. Recent data for insects support a power (allometric) rather than exponential function [6], yet we find the power function performs less well in representing growth trajectories in many marine invertebrate taxa (best fit in approx. 13% of the species). Most marine invertebrates we examine here support an exponential function, with growth proportional to mass throughout ontogeny [41], as often previously applied in aquatic [24,42] and terrestrial invertebrates [43,44]. Our findings highlight that no single universal model for growth has yet been found, either within just the marine invertebrates or indeed across animals (including marine invertebrates, insects and vertebrates).

The support for *c* = 1 in equation (4.1) for a large number of invertebrate taxa has fundamental implications when considering the MTE. This scaling exponent has been much debated [18,23,45]. However, as Krüger [41] and Glazier [46] have pointed out, in von Bertalanffy's original formulation of metabolic types [9], he suggested three different ways in which rates scaled to body mass: proportionally to surface area (2/3), mass (1) or an intermediate term. Which of these is appropriate for particular taxa and species is likely to be determined by specific life-history traits [47]. Here, we find support for a value of 1 for the majority of marine invertebrate growth rate data. Indeed, isometric scaling exponents for respiration have also been argued for pelagic invertebrates [46]. The taxa included in our study are dominated by marine pelagic invertebrates, and therefore there may be evidence of similar body mass scaling in both respiration and individual growth rates in such species.

We find the WBE to be a poor fit to marine invertebrate growth and suggest that the variable nature of growth across different taxa may explain the lack of universality in the growth form. Many marine invertebrates increase their fitness by reaching maturity rapidly; thus an exponential function may be selected for. Our work highlights problems in applying a model that was tested using vertebrate data, predominantly, to explain patterns across other diverse taxa. Future efforts to produce universal understanding of growth through ontogeny need to recognize not only the important similarities in this function, but also the critical differences that are observable.
